# Comparison of serious inhaler technique errors made by device-naïve patients using three different dry powder inhalers: a randomised, crossover, open-label study

**DOI:** 10.1186/s12890-016-0169-5

**Published:** 2016-01-14

**Authors:** Henry Chrystyn, David B. Price, Mathieu Molimard, John Haughney, Sinthia Bosnic-Anticevich, Federico Lavorini, John Efthimiou, Dawn Shan, Erika Sims, Anne Burden, Catherine Hutton, Nicolas Roche

**Affiliations:** Inhalation Consultancy Ltd, Yeadon, Leeds, West Yorkshire UK; Research in Real-Life, 5a Coles Lane, Oakington, Cambridge, UK; Academic Primary Care, Division of Applied Health Sciences, University of Aberdeen, Polwarth Building, Foresterhill, Aberdeen, AB25 2ZD UK; Department of Medical Pharmacology, CHU et Univ. de Bordeaux, Bordeaux, France; Woolcock Institute of Medical Research, Sydney Medical School, University of Sydney and Sydney Local Health District, Sydney, Australia; Department of Experimental and Clinical Medicine, Careggi University Hospital, Florence, Italy; Horizon Respiratory Consultancy, Oxford, UK; Norwich Medical School, University of East Anglia, Norwich Research Park, Norwich, UK; University Paris Descartes (EA2511), Respiratory and Intensive Care Medicine Department, Cochin Hospital Group, AP-HP, Paris, France

**Keywords:** Asthma, Chronic obstructive pulmonary disease, Serious errors, Dry powder inhaler, Inhaler technique, Mastery

## Abstract

**Background:**

Serious inhaler technique errors can impair drug delivery to the lungs. This randomised, crossover, open-label study evaluated the proportion of patients making predefined serious errors with Pulmojet compared with Diskus and Turbohaler dry powder inhalers.

**Methods:**

Patients ≥18 years old with asthma and/or COPD who were current users of an inhaler but naïve to the study devices were assigned to inhaler technique assessment on Pulmojet and either Diskus or Turbohaler in a randomised order. Patients inhaled through empty devices after reading the patient information leaflet. If serious errors potentially affecting dose delivery were recorded, they repeated the inhalations after watching a training video. Inhaler technique was assessed by a trained nurse observer and an electronic inhalation profile recorder.

**Results:**

Baseline patient characteristics were similar between randomisation arms for the Pulmojet-Diskus (*n* = 277) and Pulmojet-Turbohaler (*n* = 144) comparisons. Non-inferiority in the proportions of patients recording no nurse-observed serious errors was demonstrated for both Pulmojet versus Diskus, and Pulmojet versus Turbohaler; therefore, superiority was tested. Patients were significantly less likely to make ≥1 nurse-observed serious errors using Pulmojet compared with Diskus (odds ratio, 0.31; 95 % CI, 0.19–0.51) or Pulmojet compared with Turbohaler (0.23; 0.12–0.44) after reading the patient information leaflet with additional video instruction, if required.

**Conclusions:**

These results suggest Pulmojet is easier to learn to use correctly than the Turbohaler or Diskus for current inhaler users switching to a new dry powder inhaler.

**Trial registration:**

ClinicalTrials.gov Identifier: NCT01794390 (February 14, 2013)

**Electronic supplementary material:**

The online version of this article (doi:10.1186/s12890-016-0169-5) contains supplementary material, which is available to authorized users.

## Background

Serious inhaler technique errors made by patients with asthma and chronic obstructive pulmonary disease (COPD) are common in real life with both pressurised metered dose inhalers (pMDIs) and dry powder inhalers (DPIs) despite advances in inhaler device technology [1, 2]. Although study results vary, estimates of those making inhaler errors range up to 92 % of patients using pMDIs [3] and up to 54 % of patients using DPIs [2].

There is increasing evidence to suggest that correct inhaler technique (mastery) is fundamental for effective therapy [1, 4, 5] and that inhaler device type and mastery play important roles in improving adherence, clinical outcomes, quality of life, and use of healthcare resources [1, 6–10]. Poor inhaler technique can significantly reduce effective delivery of the respirable fraction of the emitted dose that reaches the lungs [5, 11–17]. Evidence suggests that prescribers should consider inhaler technique and ease of use before changing the dose of inhaled corticosteroids, switching to a different inhaler, or adding other treatments to the regimen of patients with poorly controlled asthma [11, 18]. Recent international asthma guidelines highlight the importance of testing and ensuring inhaler technique mastery, alongside checking adherence, before increasing or changing therapy [19].

Correct inhaler technique involves some common steps for all devices (dose preparation, device orientation, full exhalation, deep inhalation, breath hold). However, dose preparation and device orientation differ between devices, highlighting the need for tailored patient training, testing, and education [5, 6, 18, 20]. An important aspect of inhaler mastery is the absence of serious inhaler technique errors, defined as errors that could affect adequate dose delivery to the lungs (also referred to as critical or major errors [21, 22]). Innovative and reliable inhalers that are associated with a reduced risk of serious errors, as compared with current commonly used inhalers, are needed to improve effective use, adherence, and disease control [1, 5, 7, 14, 15, 23].

**Fig. 1:**
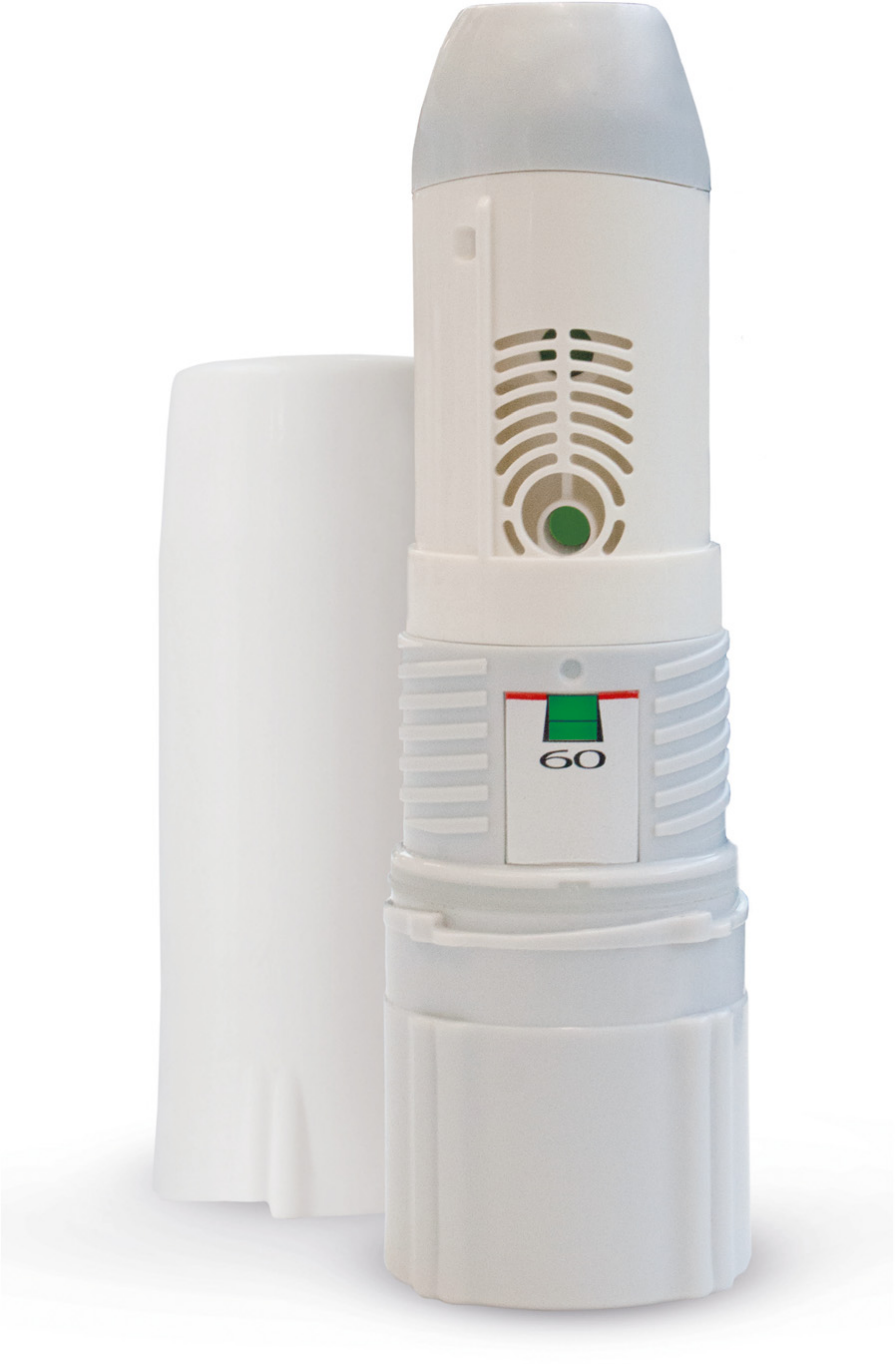
The Pulmojet inhaler

The Pulmojet®^1^ inhaler, shown in Fig. 1, is a new, prefilled DPI device that has been designed to minimise the likelihood of serious errors by reducing the number of steps required to prepare the dose. It has been designed with a mechanism that releases the dose when a set inhalation flow has been achieved. This ensures efficient de-aggregation of the dose during each inhalation, and the device can be held in any position (even downwards) during dose preparation. This design may facilitate inhaler device training, shortening its required duration and frequency. The aim of this cross-sectional, randomised, open-label study was to explore the proportion of patients making serious errors during their first training session with Pulmojet (not available for prescription during the study) as compared with two other commonly used DPIs.

## Methods

### Study design and patients

**Fig. 2:**
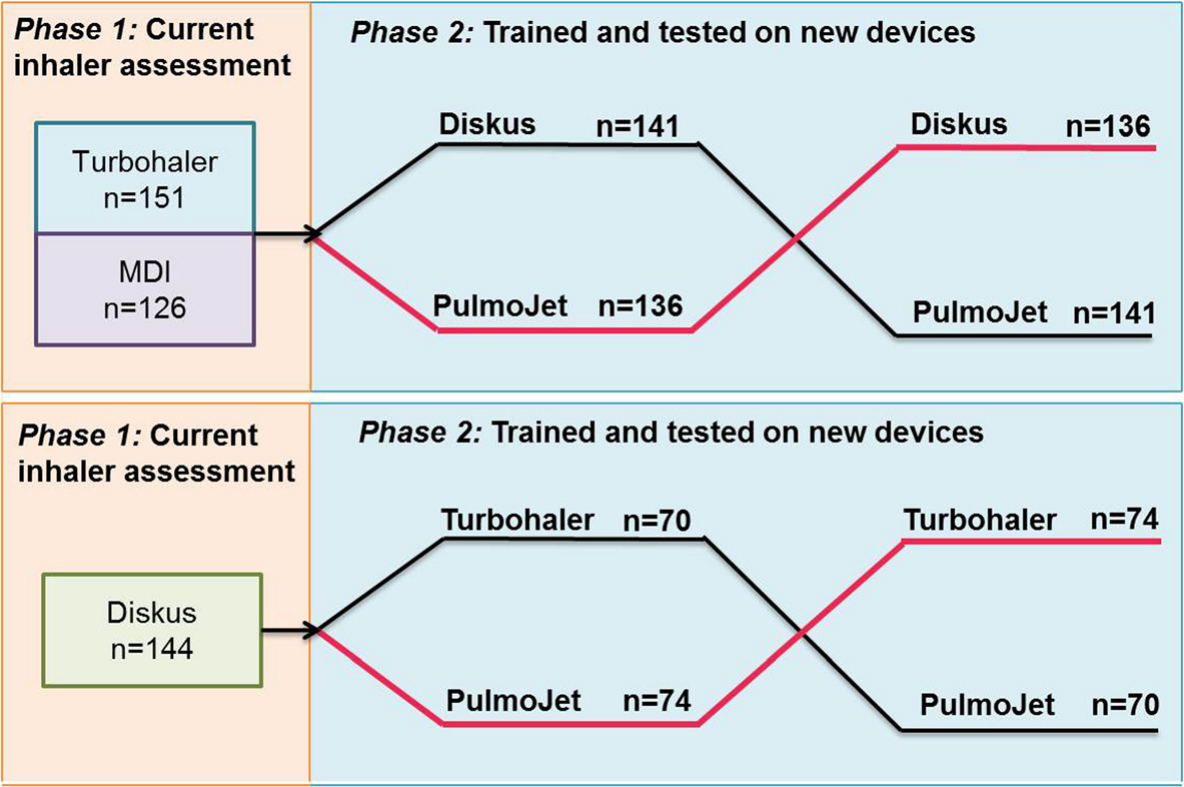
Study design showing phases 1 and 2

The Handling Inhalers – Technique Error Comparison (HI-TEC) Study was a single-visit, randomised, crossover, open-label study of patients with an established diagnosis of asthma and/or COPD, designed to evaluate serious errors in (a) the use of the patient’s own current inhaler (Turbohaler®^,^^2^ Diskus®^,^^3^ or pMDI) and (b) the first time use of a Pulmojet inhaler compared with a Turbohaler or Diskus inhaler for patients naïve to test devices (Fig. 2). The study was conducted between September 2013 and March 2014 at general practices in England and Scotland.

The study was performed in compliance with Good Clinical Practice and in accordance with the principles of the Declaration of Helsinki. It was approved by the National Research Ethics Service Cambridge East Research Ethics Committee (13/EE/0073) and by the NIHR Coordinated System for Gaining NHS Permissions (2013GP22 - 116238) (ClinicalTrials.gov Identifier: NCT01794390). In addition, local National Health Service (NHS) research governance approval was obtained from all participating practices.

To be eligible for the study, patients were required to meet the following inclusion criteria: age 18 years or older, current asthma and/or COPD physician diagnosis, current therapy including either inhaled corticosteroid or fixed-dose combination inhaled corticosteroid/long-acting beta-agonist administered through a pMDI, Turbohaler, or Diskus device, and able and willing to read and comprehend written and verbal instructions and to provide written informed consent. In addition, all patients using a Turbohaler or pMDI were required to have no use of the Diskus device in the prior year (Diskus-naïve) and all patients using a Diskus were required to have no use of the Turbohaler device in the prior year (Turbohaler-naïve). No patients had experience of Pulmojet, which remained unavailable for prescription throughout the study.

Key exclusion criteria were receipt of oral corticosteroids and/or antibiotics for a lower respiratory condition in the 4 weeks preceding the study and being considered by the study nurse to be clinically unsuitable for study inclusion for safety reasons (eg, unsafe for the patient to perform multiple inhalations for error assessments and flow measurements).

Eligible patients were identified from primary care practices using routine electronic medical record data. Patients who accepted the mailed invitation to participate in the study were scheduled for a single study visit at which their eligibility was confirmed, written informed consent was recorded, and their inhaler technique was assessed, as described below.

#### Phase 1

An electronic case report form was used to record patient demographic information and the routine clinical assessment, including the number of exacerbations in the previous 12 months, drawing on information in the patient’s medical record as necessary.

Patients were told to withhold their morning medication and to bring their current inhaler devices to the clinic. They underwent assessment of their current inhaler technique, first using their active inhaler device and assessed by trained nurse observers (using a predefined error check-list). Second, patients were then asked to repeat their inhalation manoeuvre using an empty version of their current device (containing no drug or placebo formulation), and their inhaler inhalation was assessed by an electronic inhalation profile recorder (technology assessment, described below).

#### Phase 2

Patients were allocated into the study comparison according to their current device (see Fig. 2): current pMDI and Turbohaler users were allocated to the Pulmojet-Diskus comparison and current Diskus users were allocated to the Pulmojet-Turbohaler comparison. The pMDI users were included in the Pulmojet-Diskus comparison to attain the greater numbers required according to the power calculations (see below).

The study was conducted by primary care respiratory research nurses who had prior experience and received instruction in inhaler device assessment for the study. To ensure that patient training on the different devices (Pulmojet, the investigative device, and Turbohaler or Diskus, the control devices) was consistent and representative of best standard care, two forms of patient training were utilised: provision of the device’s patient information leaflet and, for those making one or more errors post-leaflet, an instructional video. The training video was designed to be representative of a standardised form of optimal training by a qualified nurse. It was reviewed and approved by the full study steering committee.

After randomisation to device order, the first allocated device and accompanying patient information leaflet were provided to patients; they were given up to 5 min to read the leaflet after which they were evaluated for device handling technique, first by the research nurse, as evaluated against a predefined list of errors, and then via pneumotrac spirometer (details are below). If the research nurse observed no serious errors, testing on the first device was considered to have been completed and the patient was given the second allocated device. If one or more serious errors were observed in the patient’s handling of the first allocated device, the patient continued to video training and their technique was re-evaluated using both nurse-observed and technology assessments. They then repeated the same process for the second device.

### Inhaler technique assessment

Inhaler technique was evaluated by both (a) a nurse observer qualified to identify serious errors in device handling technique (see Additional file 1 for the list of predefined errors) and (b) the measurement of an inhalation profile (technology assessment of the inhalation manoeuvre). Inhalation flow against time measurements were downloaded using the inhaler attached to the inlet of a pneumotrac spirometer (Vitalograph Ltd, Maids Moreton, Buckingham, UK) so that patients were instructed to inhale through the spirometer as though through the inhaler device, as previously described [24].

At the end of the study visit, which lasted 1 h, and only after the study device training was completed, patients who demonstrated errors in current device handling were retrained by the study nurse to use their current device correctly.

All devices were empty and since no active medication was administered, efficacious and safe use were not assessed.

### Statistical analysis

#### Determination of sample size

The objective of the analyses was to demonstrate non-inferiority, and, if met, to evaluate for superiority of the Pulmojet device compared with Diskus and Turbohaler in terms of ability to achieve inhaler mastery after standardised training.

The sample sizes were calculated using nQuery Advisor 7.0 (Statistical Solutions, Ltd., Cork, Ireland) and were optimised to maximise power for the primary comparisons (see Additional file 2 for details). Based on primary care audit data from 336 patients, we estimated a success rate of 78.2 % for the Diskus inhaler and a success rate of 38.8 % for Turbohaler. We further estimated the proportion of discordant pairs in the Diskus-naïve vs. Pulmojet comparison to be 0.282 and the proportion of discordant pairs in the Turbohaler-naïve vs. Pulmojet comparison to be 0.563. To test for non-inferiority, the sample size required for the Diskus-naïve vs. Pulmojet comparison to achieve 90 % power (5 % level of significance, one-sided test) was 226, assuming an expected difference in proportions of 0.00 and allowing a difference in proportions (Pulmojet-Diskus) no lower than −0.10. Thus, it was determined that 113 patients would be randomised to Pulmojet first and a further 113 to Diskus first. For the Turbohaler-naïve vs. Pulmojet comparison, the sample size required to achieve 90 % power (5 % level of significance, one-sided test) was 122, assuming an expected difference in proportions of 0.10 and allowing a difference in proportions (Pulmojet-Turbohaler) no lower than −0.10. Sixty-one patients would, therefore, be randomised to Pulmojet and then Turbohaler and 61 to Turbohaler and then Pulmojet assessments.

The total number of patients required to achieve 90 % power was therefore 348. We anticipated an 8 % dropout rate based on a review of anonymous medical records (data not shown); thus, it was determined that 376 patients would be recruited into the study.

Subsequent to the completion of the study it was determined that the per-protocol power calculation underestimated the number of patients required. This is because the 95 % CIs (two-sided) for the non-inferiority calculation are actually equivalent to a one-sided test with 2.5 % significance. A post-hoc revised power calculation, using the assumptions noted above, and 2.5 % significance level, would have required 276 patients for the Pulmojet-Diskus comparison and 148 patients for the Pulmojet-Turbohaler comparison. As noted below, in fact the numbers of eligible patients included in the analyses were 277 and 144, respectively (421 total), which essentially also met the 95 % CIs (two-sided) for the non-inferiority calculation and one-sided test with 2.5 % significance needs of the post-hoc power calculation.

#### Baseline data

Baseline data were analysed for all enrolled patients and for the full analysis set, which included all patients who completed an assessment of nurse-observed errors on both study devices. Descriptive statistics were used to summarise patient demographic and baseline clinical characteristics. Current device arms were compared using parametric or non-parametric tests, as appropriate: for variables measured on the interval or ratio scale, an F-test or Kruskal-Wallis test (depending on the distribution of the variable) was used; for categorical variables, a Pearson’s χ^2^ (or Fisher’s exact test if cell sizes were sufficient) was used. Randomisation orders were compared for patient baseline characteristics using the Mann–Whitney and χ^2^ test.

#### Outcome measures

All analyses were carried out using the full analysis set, thus permitting a paired analysis of results.

#### Primary endpoint

To determine *non-inferiority* in device handling of the Pulmojet compared with the comparator device (Diskus or Turbohaler), the proportions of patients achieving an absence of serious errors on each device, and the difference in proportions, were analysed using a conditional binary logistic regression model. Non-inferiority in device handling was considered to have been achieved if the proportion of patients recording an absence of nurse-observed serious errors on the Pulmojet was no more than 10 % lower than the proportion of patients recording an absence of nurse-observed serious errors on the comparator device, namely, if the lower bound of the 95 % CI of the difference in proportions of patients recording an absence of serious errors was > −0.10.

Where non-inferiority was demonstrated, *superiority* was evaluated and claimed if the proportion of patients recording no nurse-observed serious errors on the Pulmojet was significantly greater than the proportion of patients recording no nurse-observed serious errors on the comparator device, namely, if the lower bound of the 95 % CI of the difference in proportions of patients recording no serious errors was >0.00. To provide an additional measure of effect size, a conditional logistic regression was used to compare the odds of recording a serious error. Superiority was also shown if the 95 % CI for the odds ratio of recording an error using Pulmojet compared with the comparator device fell entirely to the left of 1.00. The number of nurse-observed inhaler technique errors was summarised and compared between inhaler types using the Wilcoxon matched pair signed-rank test.

#### Post-hoc sensitivity analysis

A post-hoc sensitivity analysis restricted to errors that were definitely serious (ie, those that would definitely preclude adequate dose delivery to the lungs) was also conducted.

#### Secondary endpoint

Technology-assessed inhalation errors (defined as errors recorded by the pneumotrac spirometer) were combined with nurse-observed serious errors (excluding inhalation errors).

Statistically significant results were defined as *p* < 0.05. All analyses were carried out using SPSS version 22, SAS version 9.3, and Microsoft Office EXCEL 2007.

## Results

### Patients

**Fig. 3:**
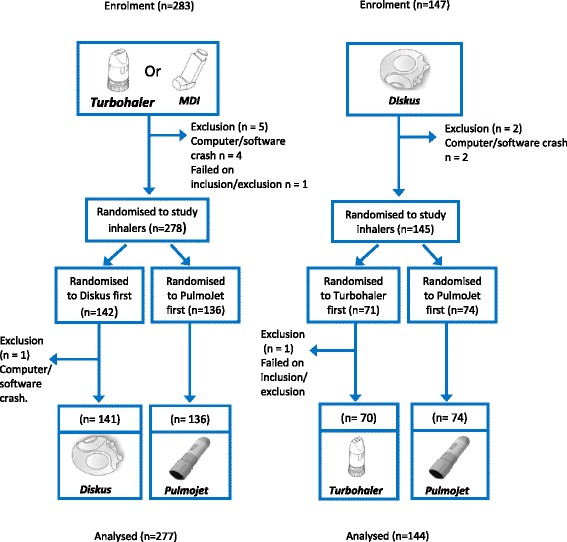
Disposition of study patients

Figure 3 shows the disposition of patients recruited into the study. Although the planned recruitment was 376 patients, 430 patients were actually recruited because of simultaneous recruitment across multiple sites. Of 430 recruited patients, 421 (98 %) completed the study and were included in the full analysis set (all patients who completed an assessment of nurse-observed errors on both study devices).

Table 1 presents baseline patient characteristics for the Diskus-naïve vs. Pulmojet-naïve and Turbohaler-naïve vs. Pulmojet-naïve comparisons. Patients first randomised to Turbohaler in the Pulmojet vs. Turbohaler comparison had received significantly more courses of oral corticosteroids in the 12 months before entry into the study than did those first randomised to Pulmojet. There were no other statistically significant differences between treatment cohorts (Table 1).

**Table 1:** Baseline patient characteristics

Characteristic	Pulmojet vs. Diskus		Pulmojet vs. Turbohaler	
	First randomised device		First randomised device	
	Pulmojet	Diskus	Pulmojet	Turbohaler
	(*n* = 136)	(*n* = 141)	(*n* = 74)	(*n* = 70)
Female sex, n (%)	89 (65.4)	91 (64.5)	32 (43.2)	35 (50.0)
Age, mean (SD)	52.5 (10.9)	51.4 (10.5)	60.7 (10.1)	59.5 (11.7)
BMI (kg/m^2^), mean (SD)	29.5 (6.9)	30.4 (6.6)	29.4 (7.3)	28.1 (7.1)
Smoking status, n (%)				
Current smoker	21 (15.4)	25 (17.7)	14 (18.9)	23 (32.9)
Ex-smoker	50 (36.8)	47 (33.3)	43 (58.1)	34 (48.6)
Non-smoker	65 (47.8)	69 (48.9)	17 (23.0)	13 (18.6)
Diagnosis, n (%)				
Asthma	118 (86.8)	114 (80.9)	23 (31.1)	29 (41.4)
COPD	17 (12.5)	23 (16.3)	51 (68.9)	41 (58.6)
Asthma & COPD	1 (0.7)	4 (2.8)	0 (0)	0 (0)
FEV_1_ %predicted, mean (SD)^b^	84 (21)	84 (22)	69 (23)	70 (25)
FEV_1_/FVC, mean (SD)^b^	0.78 (0.15)	0.75 (0.15)	0.63 (0.16)	0.66 (0.17)
Oral corticosteroid courses, n (%)^a^				
1 course	21 (15.6)	17 (12.1)	11 (15.1)	22 (31.9)*
≥2 courses	21 (15.6)	14 (9.9)	15 (20.5)	17 (24.6)
Inpatient admission, n (%)^a^				
≥1 admissions	10 (7.4)	6 (4.3)	5 (6.8)	3 (4.3)
Emergency department attendance, n (%)^a^				
≥1 visits	11 (8.1)	8 (5.7)	3 (4.1)	2 (2.9)

*COPD* chronic obstructive pulmonary disease, *FEV*_*1*_ forced expiratory volume in 1 s, *FVC* forced vital capacity

*χ^2^ test *p* < 0.05 for the two-way comparison; all other comparisons were non-significant

^a^Patient-reported with regard to prior year

^b^FEV_1_ and FEV_1_/FVC data were available for 128 (94 %) and 128 (91 %) of patients first randomised to Pulmojet and Diskus, respectively, and for 71 (96 %) and 68 (97 %) patients first randomised to Pulmojet and Turbohaler, respectively

### Phase 1

Patients were first assessed for inhaler technique using their current device: 92 % of patients using a pMDI, 39 % of those using Diskus, and 76 % of those using the Turbohaler made one or more serious errors (nurse observed and technology assessed, detailed data not shown).

### Primary endpoint: nurse-observed serious errors

#### Pulmojet vs. Diskus

**Fig. 4:**
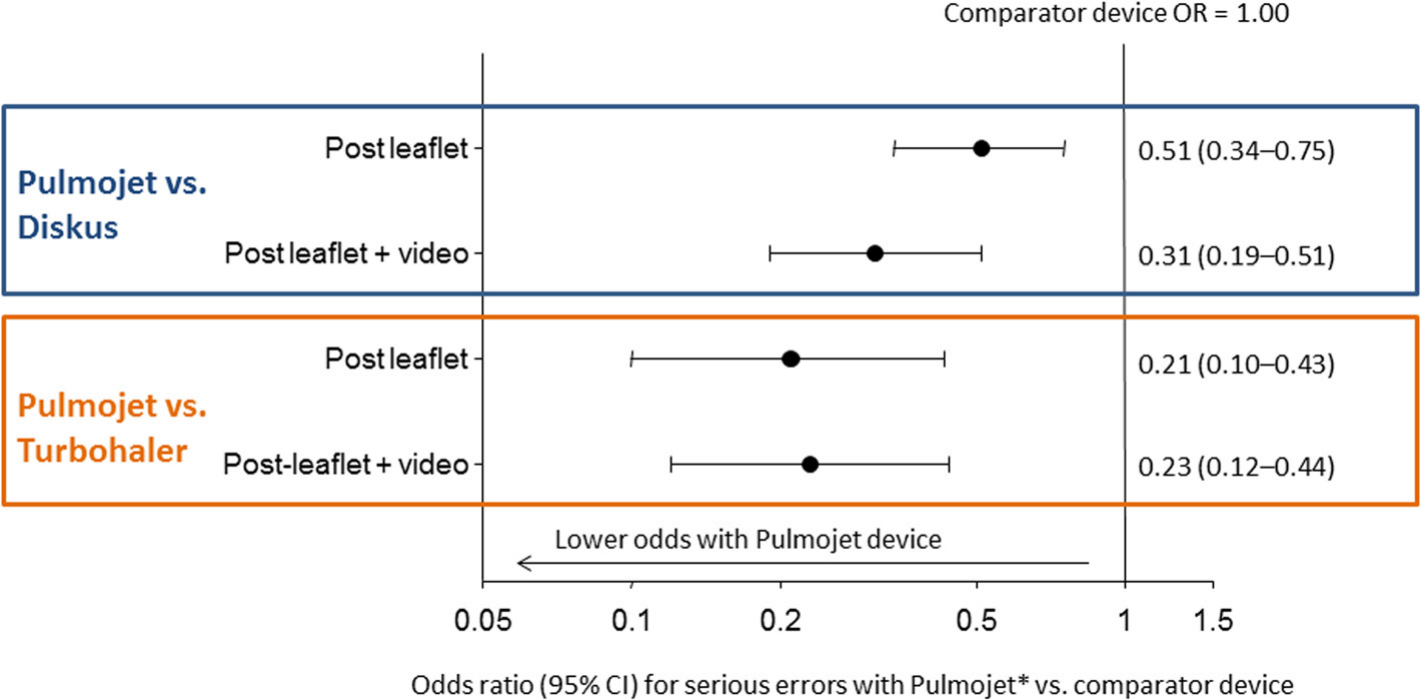
Odds ratio for ≥1 nurse-observed serious errors with Pulmojet relative to Diskus or Turbohaler DPI. (Post-leaflet + video was the primary endpoint.) *Conditional logistic regression (*p* < 0.001 for all comparisons)

Table 2 shows that more patients made errors with Diskus compared with Pulmojet and non-inferiority was found. Further analysis revealed superiority in that patients naïve to both devices had significantly lower odds of making a nurse-observed serious error (*p* < 0.001), and overall they made significantly fewer nurse-observed serious errors, when using the Pulmojet device compared with Diskus, having read the leaflet and, if required, watched an instructional video (Fig. 4; Tables 2 and 3).

**Table 2:** Nurse-observed serious errors for Diskus vs. Pulmojet and Turbohaler vs. Pulmojet comparisons: post-patient information leaflet alone and post-leaflet and instructional video

	First randomised device		Non-inferiority
Pulmojet vs. Diskus	Pulmojet	Diskus	Proportions of patients with no errors:
	(*n* = 277)	(*n* = 277)	Difference (95 % CI)
*Post-patient information leaflet alone*			
No serious error, n (%)	110 (39.7)	74 (26.7)	0.13 (0.05–0.21)
≥1 errors, n (%)	167 (60.3)	203 (73.3)	--
*Post-patient information leaflet and instructional video (primary endpoint)*			
No serious error, n (%)	215 (77.6)	170 (61.4)	0.16 (0.09–0.24)
≥1 errors, n (%)	62 (22.4)	107 (38.6)	--
Pulmojet vs. Turbohaler	Pulmojet (*n* = 144)	Turbohaler (*n* = 144)	
*Post-patient information leaflet alone*			
No serious error, n (%)	59 (41.0)	25 (17.4)	0.24 (0.13–0.34)
≥1 errors, n (%)	85 (59.0)	119 (82.6)	--
*Post-patient information leaflet and instructional video (primary endpoint)*			
No serious error, n (%)	106 (73.6)	69 (47.9)	0.26 (0.15–0.37)
≥1 errors, n (%)	38 (26.4)	75 (52.1)	--

**Table 3:** Number of patients making 0, 1, and ≥2 nurse-observed serious errors (post-patient information leaflet alone and post-leaflet and instructional video)

	First randomised device			
	Pulmojet vs. Diskus		Pulmojet vs. Turbohaler	
	Pulmojet	Diskus	Pulmojet	Turbohaler
	(*n* = 277)	(*n* = 277)	(*n* = 144)	(*n* = 144)
*Post-patient information leaflet alone*				
0 errors, n (%)	110 (39.7)	74 (26.7)	59 (41.0)	25 (17.4)
1 error, n (%)	82 (29.6)	76 (27.4)	40 (27.8)	24 (16.7)
≥2 errors, n (%)	85 (30.7)	127 (45.8)	45 (31.3)	95 (66.0)
*Post-patient information leaflet and instructional video*				
0 errors, n (%)	215 (77.6)	170 (61.4)	106 (73.6)	69 (47.9)
1 error, n (%)	43 (15.5)	64 (23.1)	26 (18.1)	36 (25.0)
≥2 errors, n (%)	19 (6.9)	43 (15.5)	12 (8.3)	39 (27.1)

**Fig. 5:**
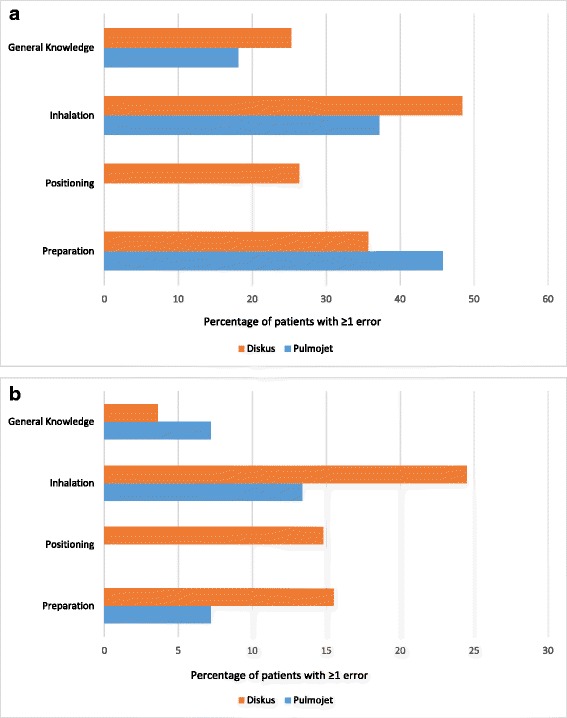
**a**. Percentage of patients recording Pulmojet and Diskus errors post-leaflet alone. **b**. Percentage of patients recording Pulmojet and Diskus errors post-leaflet and instructional video

The post-leaflet nurse-observed serious errors categorised into preparation, positioning, inhalation, and general knowledge errors are presented in Fig. 5a (see Additional file 1 for further details). More patients made more general knowledge and inhalation errors with Diskus than with Pulmojet, whereas more patients made preparation errors with Pulmojet than with Diskus. In addition, 26 % of patients made a positioning error with the Diskus. Figure 5b shows that post-leaflet and post-leaflet + video, more patients made inhalation and preparation errors with Diskus compared with Pulmojet; in addition, there were positioning errors with the Diskus. As the Pulmojet device is not subject to positioning error, no positioning errors were identified with Pulmojet. On the other hand, more patients did not know how to determine when the device was empty, which was considered a general knowledge error, with Pulmojet than with Diskus (details in Additional file 1: Tables S1 and S2).

The numbers of patients making 0, 1, and ≥2 serious errors are summarised in Table 3.

#### Pulmojet vs. Turbohaler

Table 2 and Fig. 4 show non-inferiority and also superiority in that fewer patients made nurse-observed serious errors with Pulmojet compared with Turbohaler after the leaflet and video instruction, as well as the leaflet alone. Many patients made more than one error, particularly post-leaflet alone (Table 3).

**Fig. 6:**
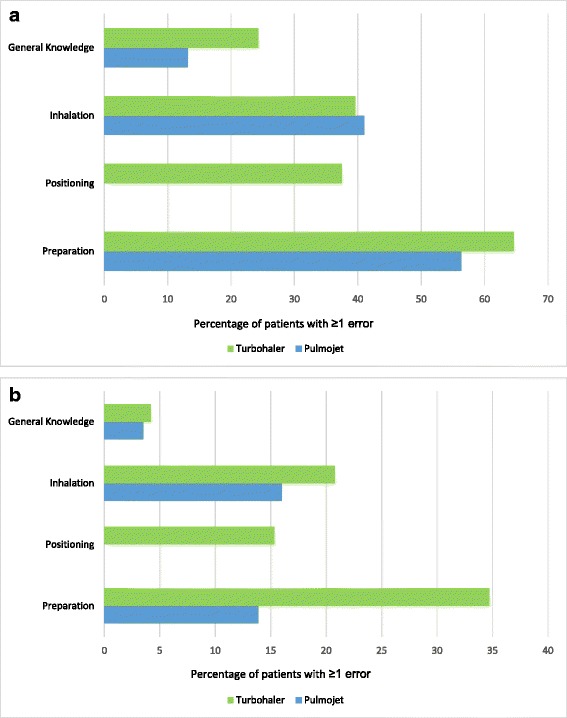
**a**. Percentage of patients recording Pulmojet and Turbohaler errors post-leaflet alone. **b**. Percentage of patients recording Pulmojet and Turbohaler errors post-leaflet and instructional video

Serious errors by type are shown in Fig. 6a and b. In addition to making positioning errors with Turbohaler, more patients made general knowledge, inhalation, and preparation errors with Turbohaler than with Pulmojet (Additional file 1: Tables S3 and S4).

### Post hoc sensitivity analysis

The sensitivity analysis examining errors that were definitely serious (those that would definitely preclude adequate dose delivery to the lungs; see Additional file 1: Tables S1–S4) found that non-inferiority remained for both comparisons (Pulmojet vs. Diskus and Pulmojet vs. Turbohaler), post-leaflet alone and post-leaflet and instructional video (Table 4).

**Table 4:** Nurse-observed, definitely serious errors for Diskus vs. Pulmojet and Turbohaler vs. Pulmojet comparisons: post-patient information leaflet alone and post-leaflet and instructional video (post-hoc sensitivity analysis)

	First randomised device		Non-inferiority	Superiority	
Pulmojet vs. Diskus	Pulmojet	Diskus	Proportions of patients with no errors: Difference (95 % CI)	Serious error with Pulmojet^a^ relative to comparator (1.00): Odds ratio (95 % CI)	*p-value* ^a^
	(*n* = 277)	(*n* = 277)			
*Post-patient information leaflet alone*					
No definitely serious error, n (%)	147 (53.1)	129 (46.6)	0.06 (−0.02 to 0.15)	--	--
≥1 errors, n (%)	130 (46.9)	148 (53.4)	--	0.74 (0.51–1.06)	0.099
*Post-patient information leaflet and instructional video*					
No definitely serious error, n (%)	229 (82.7)	206 (74.4)	0.08 (0.01–0.15)	--	--
≥1 errors, n (%)	48 (17.3)	71 (25.6)	--	0.45 (0.26–0.78)	0.004
Pulmojet vs. Turbohaler	Pulmojet (*n* = 144)	Turbohaler (*n* = 144)			
*Post-patient information leaflet alone*					
No definitely serious error, n (%)	78 (54.2)	44 (30.6)	0.24 (0.13–0.35)	--	--
≥1 errors, n (%)	66 (45.8)	100 (69.4)	--	0.26 (0.14–0.49)	<0.001
*Post-patient information leaflet and instructional video*					
No definitely serious error, n (%)	115 (79.9)	85 (59.0)	0.21 (0.10–0.31)	--	--
≥1 errors, n (%)	29 (20.1)	59 (41.0)	--	0.32 (0.17–0.58)	<0.001

^a^Conditional logistic regression

Post-leaflet alone, for the Pulmojet vs. Diskus comparison, superiority was no longer shown (Table 4). Overall, both preparation and inhalation errors were lower with Pulmojet than Diskus (Additional file 1: Tables S1 and S2). Post-leaflet and instructional video, superiority was shown. Preparation errors with Pulmojet were very low, while inhalation errors with Diskus remained high.

For the Pulmojet vs. Turbohaler comparison, superiority was shown both post-leaflet alone and post-leaflet and instructional video, with high preparation errors for Turbohaler in both cases (Table 4; Additional file 1: Tables S3 and S4).

### Secondary endpoint: combined serious errors (nurse-observed and technology-assessed)

Table 5 shows superiority of Pulmojet use when technology-assessed errors were combined with nurse-observed errors.

**Table 5:** Combined serious errors (nurse-observed and technology-assessed) for Diskus vs. Pulmojet and Turbohaler vs. Pulmojet comparisons: post-patient information leaflet alone and post-leaflet and instructional video

	First randomised device		Superiority	
Pulmojet vs. Diskus	Pulmojet (*n* = 272)	Diskus (*n* = 272)	Odds ratio (95 % CI) for Pulmojet^a^ relative to comparator (1.00)	*p-value* ^a^
*Post-patient information leaflet alone*				
≥1 errors, n (%)	148 (54.4)	175 (64.3)	0.61 (0.42–0.90)	0.012
*Post-patient information leaflet and instructional video*				
≥1 errors, n (%)	56 (20.6)	87 (32.2)^b^	0.48 (0.31–0.75)	0.001
Pulmojet vs. Turbohaler	Pulmojet (*n* = 144)	Turbohaler (*n* = 142)		
*Post-patient information leaflet alone*				
≥1 errors, n (%)	79 (54.9)	121 (85.2)	0.16 (0.08–0.34)	<0.001
*Post-patient information leaflet and instructional video*				
≥1 errors, n (%)	38 (26.4)	93 (65.5)	0.10 (0.04–0.23)	<0.001

^a^Conditional logistic regression

^b^ *n* = 270

## Discussion

Results of the current study suggest that Pulmojet may be easier to learn to use correctly than either the Diskus or Turbohaler for current inhaler users who are switched to a new device. Patients with asthma and/or COPD were significantly less likely to make a nurse-observed serious error with Pulmojet than with Diskus or Turbohaler after receiving training by either the patient information leaflet alone or the leaflet and as-needed additional video instruction. Mastery of the inhalation technique is, therefore, more likely to be readily achieved with Pulmojet than either Diskus or Turbohaler. Designing inhaler devices to make them easier to use and teach is essential for improving long-term outcomes in asthma and COPD, so an important factor to consider when choosing a specific device is whether or not the patient is capable of using it correctly to achieve adequate drug delivery [25]. Furthermore, the ease of correct use of inhalers and patient preference for device can affect adherence with treatment and clinical outcomes [1, 5, 7, 14–16, 23–26].

Patients were not given verbal instructions alongside the patient information leaflet, so the use of the leaflet on its own mimicked common clinical practice (although sometimes patients may be verbally trained as well when prescribed a new device). We found that, for all inhalers tested, fewer serious errors were observed after patients read the patient information leaflet and watched an instructional video than after reading the leaflet alone. Our findings are in line with other reports that written or passive instructions alone, such as the patient information leaflets, are not sufficient to teach correct inhalation technique; and other tools, such as verbal instructions, multimedia educational materials, demonstrations, and practice sessions are needed to improve inhaler device technique [25–35]. Crompton et al. [26] have also recommended that the teaching of correct inhalation techniques should be tailored to each patient’s needs and preferences; for example, multimedia methods may be more beneficial for younger patients, while one-to-one tuition is more suitable for elderly patients. The fact that errors in device technique remained after the instructional video suggests that instructional videos are useful but may not be adequate to ensure optimal device technique for all patients.

The comparator devices chosen for this study, the Diskus and the Turbohaler, are two of the most frequently prescribed multidose DPI devices worldwide. Previously reported incidence of serious errors ranges from 21–35 % with Diskus and 37–44 % with Turbohaler [1, 10]. The current device error data (study phase 1) showed a similar incidence for Diskus use (39 %) but greater error rates amongst the Turbohaler users (76 %). The high inhaler technique error rate amongst current pMDI users (92 %) was consistent with previous data [3].

The Pulmojet device has been designed to release its dose at a low minimum inspiratory flow. In-house measurements have identified that the dose is released from a Pulmojet at ≥25 L/min (unpublished data), which is below the recognised minimum inspiratory flow of the Turbohaler [36–38] and the Diskus [39]. This ensures efficient de-aggregation of the dose during each inhalation so that drug particles most likely to be deposited into the airways are entrained in the inhaled airstream leaving the device. The Pulmojet inhaler has been designed with audio, visual, and sensory feedback mechanisms to indicate that an adequate inhalation has been performed, a feature appreciated by patients [40], whereas neither the Turbohaler nor Diskus provides any feedback to the patient that an adequate inspiratory flow has been achieved. Furthermore, because the dose is not released until a set inhalation flow is achieved, the Pulmojet can be held in any orientation during dose preparation.

Patients attending UK primary care practices were recruited for this study, and minimal exclusion criteria other than evidence of recent exacerbation or lower respiratory tract infection were applied to enable the study results to be generalisable to most patients with asthma and/or COPD receiving ICS or fixed-dose combination therapy in routine practice. Asthma, COPD and asthma-COPD were diagnoses made by the patients’ physicians according to their standard clinical practices, and we included both ICS and fixed-dose combinations as both are commonly prescribed for asthma and COPD in clinical practice [41]. Other strengths of this investigation include the randomised assignment to device training order, assessments made by trained independent nurses, and blinding of data analysts and statisticians. The practical importance of identifying superiority (rather than just non-inferiority) should also be recognised, as despite the ease of use of any one device compared with another, the overall management of chronic respiratory conditions is associated with a complex array of management issues, and hence it would be practically acceptable for a switch in device only if superiority were shown.

We acknowledge several limitations of the current research. Firstly, this was an open-label study that involved subjective assessment, and hence potential nurse bias, although efforts were made to standardise demonstration of devices and training in assessment of serious errors. Secondly, this study focused on self-training techniques (both the patient information leaflet and video are tools that the patient can employ at home); whereas, in practice, healthcare providers might incorporate their own educational style when training device-naïve patients for the first time (ie, the education may not be standardised in real life). Furthermore, although errors were defined by independent expert consensus, they were not validated as being serious. However, in the post-hoc sensitivity analysis restricted to errors that were definitely serious (ie, those that would definitely preclude adequate dose delivery to the lungs), the results remained largely the same. We included “inhalation is not forceful from the start” as a serious error based on the recommendations for DPI use of a recent European Respiratory Society/International Society for Aerosols in Medicine task force [42].

Another limitation is the fact that this study does not provide insight into maintenance of inhaler mastery or its impact on disease control. Learning to correctly handle a device is a continuous process and inhaler technique can decline over time [34], so it would be interesting to determine whether the superior results in inhaler technique observed with Pulmojet vs. Diskus and Turbohaler are maintained after a period of use and whether this is associated with better disease control. Moreover, patients considered as first-time users with regard to the Diskus or Turbohaler devices could potentially have used them in the past, more than 1 year before their inclusion in the study. In such cases, it is possible that any differences with the Pulmojet device would have been minimised or, conversely, that for patients switched at some time preceding the prior year from Diskus or Turbohaler because they could not handle it well, a comparison with Pulmojet would lead to a relatively favourable outcome for Pulmojet. However, we believe that patients using these devices previous to the 1 year were few if any. Finally, all enrolled patients were current inhaler users and hence our findings may not apply to patients prescribed an inhaler for the first time.

## Conclusions

In patients with asthma and/or COPD who were given inhaler devices without proper training, fewer errors were made when using the Pulmojet for the first time when compared with Diskus and Turbohaler devices. These findings suggest that Pulmojet is a device that is easier to teach, easier to learn to use correctly, and easier to use. Thus, device mastery is more likely when using Pulmojet compared with Diskus or Turbohaler. The improvements after patients watched an instructional video suggest that videos could be useful for some individuals to complement inhaler technique training. In addition, the study design used here provides an adequate framework for future studies aimed at comparing the ease of effective training and use of other inhaler devices.

## Additional files

Additional file 1:
**Provides a list of predefined errors and percentages of patients making errors with each inhaler device.** (PDF 101 kb) Additional file 2:
**Provides details of sample size determination.** (PDF 288 kb) Additional file 3:
**Provides a list of local Principal Investigators responsible for the conduct of the study at general practice sites participating in the study.** (PDF 24 kb)

## Abbreviations

BMI: body mass index
CI: confidence interval
COPD: chronic obstructive pulmonary disease
DPI: dry powder inhaler
HI-TEC: Handling Inhalers – Technique Error Comparison
ICS: inhaled corticosteroid
NHS: National Health Service
pMDI: pressurised metered dose inhaler
SAS: Statistical Analysis System
SD: standard deviation
SPSS: Statistical Package for the Social Sciences
UK: United Kingdom

## Footnotes

1 Pulmojet® is a registered trademark in the property of Zentiva (Zentiva Group, a.s., Prague, Czech Republic)

2 Turbohaler® is a registered trademark of the AstraZeneca group of companies

3 Diskus® is a registered trademark of the GSK group of companies
