# Supplementary material for: Comparison of serious inhaler technique errors made by device-naïve patients using three different dry powder inhalers: a randomised, crossover, open-label study
Source: BMC Pulm Med. 2016 Jan 14;16:12. doi: 10.1186/s12890-016-0169-5 (PMC4712500; doi:10.1186/s12890-016-0169-5)
Supplement: Additional file 1: — Provides a list of predefined errors and percentages of patients making errors with each inhaler device. (PDF 101 kb) [file 12890_2016_169_MOESM1_ESM.pdf]

Additional file 1

Table S1 Primary endpoint: breakdown of Pulmojet errors – post-leaflet alone, and post-leaflet + video, nurse-observed serious errors, Pulmojet-Diskus comparison

Pulmojet serious errors	Error type	Post-leaflet alone (n=277), n (%)	Post-leaflet + video (n=277), n (%)
Does not remove cap*	Preparation	6 (2.2)	1 (0.4)
Green window (dose actuator) not checked	Preparation	60 (21.7)	9 (3.2)
Places fingers or mouth completely over holes in the device body (restricting airflow)*	Preparation	14 (5.1)	2 (0.7)
Fails to put in mouth and seal lips around mouthpiece*	Preparation	11 (4.0)	2 (0.7)
Inhalation is not as fast as possible from the start (defined as audible 'soft plop' heard during the first half of inhalation)*	Inhalation	85 (30.7)	37 (13.4)
Cap is not securely screwed down tightly onto base (with a snug fit) and an audible click is not heard*	Preparation	70 (25.3)	9 (3.2)
Failure to inhale through mouthpiece*	Inhalation	10 (3.6)	4 (1.4)
Inhalation through the nose at the same time as through the mouth	Inhalation	1 (0.4)	1 (0.4)
Ask patient how they know when their device is empty - Does not know	General knowledge	50 (18.1)	20 (7.2)

*Errors included in the sensitivity analysis of definitely serious errors.

Table S2 Primary endpoint: breakdown of Diskus errors - post-leaflet alone, and post-leaflet + video, nurse-observed serious errors, Pulmojet-Diskus comparison

**Diskus serious errors**	**Error type**	**Post-leaflet alone (n=277), n (%)**	**Post-leaflet + video (n=277), n (%)**
Does not slide cover as far as possible*	Preparation	33 (11.9)	12 (4.3)
Does not slide lever fully to open mouthpiece*	Preparation	63 (22.7)	23 (8.3)
Holds in a downward position after dose preparation (before an inhalation)	Positioning	73 (26.4)	41 (14.8)
Shakes after dose preparation*	Preparation	2 (0.7)	2 (0.7)
Blowing into the device before inhalation*	Preparation	21 (7.6)	6 (2.2)
Fails to put in mouth and seal lips around mouthpiece*	Preparation	17 (6.1)	10 (3.6)
Inhalation is not as fast as possible from the start (defined as a very fast suck) / Inhalation is not forceful from the start*	Inhalation	115 (41.5)	60 (21.7)
Failure to inhale through mouthpiece*	Inhalation	34 (12.3)	12 (4.3)
Inhalation through the nose at the same time as through the mouth	Inhalation	0 (0)	0 (0)
Ask patient how they know when their device is empty - Does not know.	General Knowledge	70 (25.3)	10 (3.6)

*Errors included in the sensitivity analysis of definitely serious errors.

Table S3 Primary endpoint: breakdown of Pulmojet errors - post-leaflet alone, and post-leaflet + video, nurse-observed serious errors, Pulmojet-Turbohaler comparison

**Pulmojet serious errors**	**Error type**	**Post-leaflet alone (n=144), n (%)**	**Post-leaflet + video (n=144), n (%)**
Does not remove cap*	Preparation	4 (2.8)	0 (0)
Green window (dose actuator) not checked	Preparation	51 (35.4)	11 (7.6)
Places fingers or mouth completely over holes in the device body (restricting airflow)*	Preparation	9 (6.3)	3 (2.1)
Fails to put in mouth and seal lips around mouthpiece*	Preparation	6 (4.2)	1 (0.7)
Inhalation is not as fast as possible from the start (defined as audible 'soft plop' heard during the first half of inhalation)*	Inhalation	44 (30.6)	21 (14.6)
Cap is not securely screwed down tightly onto base (with a snug fit) and an audible click is not heard*	Preparation	30 (20.8)	6 (4.2)
Failure to inhale through mouthpiece*	Inhalation	7 (4.9)	3 (2.1)
Inhalation through the nose at the same time as through the mouth	Inhalation	2 (1.4)	2 (1.4)
Ask patient how they know when their device is empty - Does not know	General knowledge	19 (13.2)	5 (3.5)

*Errors included in the sensitivity analysis of definitely serious errors.

Table S4 Primary endpoint: breakdown of Turbohaler errors - post-leaflet alone, and post-leaflet + video, nurse-observed serious errors, Pulmojet-Turbohaler comparison

**Turbohaler serious errors**	**Error type**	**Post-leaflet alone (n=144), n (%)**	**Post-leaflet + video (n=144), n (%)**
Does not remove cap*	Preparation	1 (0.7)	0 (0)
Places fingers or mouth completely over holes in the device body (restricting airflow)	Preparation	24 (16.7)	9 (6.3)
Fails to put in mouth and seal lips around mouthpiece*	Preparation	5 (3.5)	0 (0)
Shakes during preparation*†	Preparation	2 (1.4)	0 (0)
Device not held upright (mouthpiece skywards) when the base is twisted during dose preparation*‡	Preparation	55 (38.2)	31 (21.5)
Dose not prepared correctly- twisting the base until it clicks*	Preparation	31 (21.5)	14 (9.7)
Dose not prepared correctly- turn it back to the original position*	Preparation	50 (34.7)	21 (14.6)
Device not held upright (mouthpiece skywards) after the base is twisted until inhalation*‡	Positioning	54 (37.5)	22 (15.3)
Shakes after dose preparation*	Preparation	1 (0.7)	0 (0)
Exhales into the inhaler before taking dose*	Inhalation	0 (0)	0 (0)
Inhalation is not as fast as possible from the start (defined as a very fast suck) / Inhalation is not forceful from the start*	Inhalation	55 (38.2)	30 (20.8)
Failure to inhale through mouthpiece*	Inhalation	6 (4.2)	3 (2.1)
Inhalation through the nose at the same time as through the mouth	Inhalation	2 (1.4)	1 (0.7)
Cap is not securely screwed down tightly onto base	Preparation	7 (4.9)	2 (1.4)
Ask patient how they know when their device is empty - Does not know	General knowledge	35 (24.3)	6 (4.2)

*Errors included in the sensitivity analysis of definitely serious errors.

†Error based on in vitro data (unpublished).

‡Up to a 45-degree angle was allowed for inhaler positioning.
