# Supplementary material for: Comparison of serious inhaler technique errors made by device-naïve patients using three different dry powder inhalers: a randomised, crossover, open-label study
Source: BMC Pulm Med. 2016 Jan 14;16:12. doi: 10.1186/s12890-016-0169-5 (PMC4712500; doi:10.1186/s12890-016-0169-5)
Supplement: Additional file 2: — Provides details of sample size determination. (PDF 288 kb) [file 12890_2016_169_MOESM2_ESM.pdf]

Additional file 2

Supplementary information

**Comparison of serious inhaler technique errors made by device-naïve patients using three different dry powder inhalers: a randomised, crossover, open-label study**

Chrystyn H et al.

## Determination of sample size

Sample sizes were calculated using nQuery Advisor 7.0 (Statistical Solutions, Ltd., Cork, Ireland).

Optimum Patient Care Ltd (OPC) provides asthma and COPD clinical review services for primary care. This includes evaluation and remedial correction, as necessary, of current inhaler device technique. An audit of the inhaler technique for patients using Diskus and Turbohalers (n=336) showed that of patients currently using a Diskus inhaler, 78.2% had good inhaler technique (21.8% had at least 1 serious error requiring remedial correction) and of patients currently using a Turbohaler, 38.8% had good inhaler technique (61.2% had at least 1 serious error requiring remedial correction).

The proportion of discordant pairs for the PulmoJet vs Diskus comparison for a crossover study design was estimated to be 0.262 [1,2]. (Investigation of concordant vs. discordant pairs is a means of describing the relationship between pairs of observations. A discordant pair is a pair of two-variable observations in which the association between two ordinal variables goes in opposite directions.) To calculate the concordant and discordant pairs, the data are treated as ordinal, so ordinal data were appropriate for our application. The number of concordant and discordant pairs are used in calculations for Kendall's tau, which measures the association between two ordinal variables.

For the Pulmojet vs. Turbohaler comparison, the proportion of discordant pairs for a crossover study design was estimated to be 0.563. This was based on the assumption that the proportion of patients having adequate Pulmojet inhaler technique would be the same as the proportion having adequate inhaler technique using the Diskus (78.2%), as the Diskus is considered clinically to be the most similar device available.

Therefore, testing for non-inferiority (used a paired test for proportions), the following power calculations were performed.

## Phase 2 primary comparisons

***Diskus vs. Pulmojet (current Turbohaler and MDI patients)***
With a sample size of 226, a paired test with a 0.050 one-sided significance level would have 90% power to reject the null hypothesis that the proportions were not equivalent (i.e. based on an expected success rate of 78.2% for Diskus patients, the difference in proportions of Pulmojet - Diskus would fall no lower than 10%) when the expected difference in proportions is 0.000, assuming that the proportion of discordant pairs was 0.262. It was thus calculated that 113 patients would therefore be randomised to Diskus followed by Pulmojet, and 113 patients would be randomised to Pulmojet followed by Diskus.

***Turbohaler (naïve) vs. Pulmojet (current Diskus patients)***
With a sample size of 122, a paired test with a 0.050 one-sided significance level would have 90% power to reject the null hypothesis that the proportions were not equivalent (i.e. based on an expected success rate of 38.8% for Turbohaler patients, the difference in proportions of Pulmojet - Turbohaler would fall no lower than 10%) when the expected difference in proportions is 0.10, assuming that the proportion of discordant pairs was 0.563. It was thus calculated that 61 patients would therefore be randomised to Turbohaler followed by Pulmojet and 61 patients would be randomised to Pulmojet followed by Turbohaler.

The number of patients required to achieve 90% power in the phase 2 comparisons was 348 (please see the section below). Based on OPC's asthma review service data (June 2011 to February 2013), of 1869 participating patients, 90 (4.8%) failed to complete their inhaler assessments (assessments were carried out on the patient's current inhaler only). As the HI-TEC study involved assessment on three inhalers, a higher patient drop-out rate was therefore anticipated. Therefore, a planned recruitment of 376 patients into the HI-TEC study was determined to allow for an 8% patient dropout rate.

**Phase 1 (secondary) comparisons**
The following sample size numbers were required to achieve >80% power in the phase 1 comparisons.

- Current Turbohaler patients vs. Pulmojet (80 patients, 84% power)
- Current Diskus patients vs. Pulmojet (154 patients, 85% power)
- Current MDI patients vs. Pulmojet (162 patients, >90% power)

However, the study design and sample size (376 patients) restricted the number of patients available for the exploratory phase 1 (secondary) comparisons. The sample size was optimised to maximise power in the phase 2 (primary) comparisons. Consequently, the number of patients available for each phase 1 comparison is given below:

- Current Diskus patients vs. Pulmojet (122 patients)

- Current Turbohaler patients vs. Pulmojet (140 patients)
- Current MDI patients vs. Pulmojet (114 patients)

## References

1. Campbell MK, Machin D. Medical statistics: a commonsense approach. 2nd ed. Chichester: John Wiley; 1993.

2. Altman DG. Practical statistics for medical research. 1st ed. London: Chapman & Hall; 1991.
