# Supplementary material for: Comparison of serious inhaler technique errors made by device-naïve patients using three different dry powder inhalers: a randomised, crossover, open-label study
Source: BMC Pulm Med. 2016 Jan 14;16:12. doi: 10.1186/s12890-016-0169-5 (PMC4712500; doi:10.1186/s12890-016-0169-5)
Supplement: Additional file 3: — Provides a list of local Principal Investigators responsible for the conduct of the study at general practice sites participating in the study. (PDF 24 kb) [file 12890_2016_169_MOESM3_ESM.pdf]

Additional file 3

Table S5 Local Principal Investigators responsible for the conduct of the study at general practice sites participating in the study.

Study Site	Principal Investigator
Station House Surgery	Dr Sarah Woodford
Westcliffe Medical Practice	Dr. Sara Humphrey
Thornton-Denholme Medical Centre	Dr Ruth Stockwell
Kilmeny Group Medical Practice	Dr. Jim Hodgson
Shipley Medical Practice	Dr Kirsty Hogg
North Leeds Medical Practice	Dr. Katherine Hickman
Dr Kilpatrick & Partners - Two Shires	Dr Shora D Montgomerie
Castle Mead Medical Centre	Dr. David Tull
North Brink Surgery	Dr. Mandeep Sira
Parson Drove Surgery	Dr. Ashish Rastogi
Wellside Surgery	Dr. Basia Uszycka
Yaxley Group Practice	Dr Bhatia Jaspreet
Buckden and Little Paxton Surgery	Dr. Kevin Brinkhurst
Papworth Surgery	Dr. Silyee Tan
Grove Surgery,	Dr Duncan Edwards
Gurney Surgery, Mile End Road Surgery Tuckswood Surgery	Dr Joanne Walsh
Magdalen Medical Practice	Dr Helen Sharp
Mundesley Medical Centre	Dr Daryl Freeman
Mount Farm Surgery	Dr Brian Ainsworth
Chesterfield Drive Surgery	Dr. Balaji Donepudi
Stowhealth	Dr. Neil Macey
The Peninsula Practice	Dr Lindsey Crocket
Framfield House Surgery	Dr. Phil Weeks
Wickham Market Medical Centre	Dr. Will Elson

S J Morris & Partners	Dr. Yvonne Webb
Lea Vale Medical Group	Dr. Paul A Singer
Dr M L & Dr P Agrawal	Dr. M Agrawal
Neville Road Surgery	Dr. Sajid Mehmood
Whitstable Medical Centre/Chestfield Medical Centre / Estuary View Medical Centre	Dr. Hilary Pinnock
Adcroft Surgery	Dr Stephen Locke
Hawthorn Medical Centre	Dr Simon Dowdeswell
Bradford Road Medical Centre	Dr Tobias Cookson
Yeo Vale Medical Practice	Dr. Matthew Hoghton
Oak Tree Surgery	Dr Rehan Symonds
Old Bridge Surgery	Dr Pascual Daza-Ramirez
The Stennack Surgery	Dr. Sarah Shaw
Brunel Medical Practice	Dr Richard Veale
Coleridge Medical Centre	Dr Lisa Gibbons
Peterhead Medical Practice	Dr. Iain Small
